# Evaluation de la prise en charge de l’érysipèle par les médecins généralistes de la ville de Marrakech

**DOI:** 10.11604/pamj.2018.29.41.13539

**Published:** 2018-01-17

**Authors:** Fatima Ihbibane, Ntini Lebi Arsène, Latifa Adarmouch, Mohamed Amine, Noura Tassi

**Affiliations:** 1 Service des Maladies Infectieuse, CHU Mohamed VI, Université Cadi Ayyad, Marrakech, Maroc; 2 Service de Médecine Communautaire et d’Epidémiologie, Faculté de Médecine et de Pharmacie de Marrakech, Université Cadi Ayyad, Marrakech, Maroc

**Keywords:** Erysipèle, dermohypodermites bactériennes, antibiothérapie, anti-inflammatoires, Erysipelas, bacterial dermohypodermites, antibiotics, anti-inflammatory drugs

## Abstract

**Introduction:**

L'érysipèle est la plus fréquente des dermohypodermites bactériennes non nécrosantes (DHBNN). L'objectif de notre travail est d'évaluer l'adéquation des connaissances des médecins généralistes avec les données de la littérature sur la prise en charge diagnostique et thérapeutique de l'érysipèle.

**Méthodes:**

Nous avons réalisé une enquête transversale à visée descriptive et analytique auprès de 167 médecins généralistes des secteurs public et privé de Marrakech du 19 Mai au 20 Octobre 2014.

**Résultats:**

Les 114 questionnaires qui nous ont été retournés ont révélé que des facteurs de risque locaux et généraux étaient souvent retrouvés en cas d'érysipèle. Le diagnostic positif des formes typiques était clinique pour 92(80,7%) médecins. La prise en charge devrait se faire en ambulatoire pour 97(85,1%), le recours à l'hospitalisation et aux examens para-cliniques ne s'avérait nécessaire que pour les formes sévères, atypiques ou compliquées. L'amoxicilline orale a été préconisée par 25 médecins (21,9%). La bi-antibiothérapie incluant une molécule antistreptococcique a été préconisée par 15(13,2%) médecins. Le recours aux anti-inflammatoires a été préconisé par 16 médecins (14%). Les préventions primaires et secondaires ont rencontré l'intérêt de nos médecins dont 108 (94,7%) ont été favorables au traitement des portes d'entrée cutanées et 53 (46,5%) à l'antibioprophylaxie à partir de la deuxième récidive.

**Conclusion:**

D'après notre étude, l'érysipèle semble relativement fréquent en pratique de ville, les éléments du diagnostic clinique devraient faire l'objet d'une vulgarisation visant à améliorer les attitudes diagnostiques et thérapeutiques de nos médecins.

## Abstract (en)

**Introduction:**

Erysipelas is the most common non necrotizing bacterial dermohypodermitis (NNBDH). This study aimed to evaluate the adequacy of general practitioners’ knowledge about literature data on the diagnostic and therapeuthic management of erysipelas.

**Methods:**

We conducted a cross-sectional descriptive and analytical survey of 167 general practitioners in the public and private sectors in Marrakech over the period from 19 May to 20 October 2014.

**Results:**

The 114 questionnaires which had been returned revealed that local and general risk factors were often reported for erysipelas. 92 (80.7%) physicians thought that positive diagnosis of common types was based on clinical examination. 97(85.1%) physicians thought that it required only out-patient service and that hospitalization and para-clinical examinations should only be performed in patients with severe, atypical or complicated erysipelas. 25 (21.9%) physicians thought that oral amoxicillin should be the gold standard therapy. 15(13.2%) physicians thought that bi-antibiotic therapy including antistreptococcique molecule should be the gold standard. 16 doctors (14%) advocated anti-inflammatory drugs. The primary and secondary prevention levels generated interest from physicians of whom 108 (94.7%) were favorable to the treatment of the portals of entry in the skin while 53 (46.5%) to the antibioprophylaxis after the second recurrence.

**Conclusion:**

Our study highlights that erysipelas is relatively frequent in city medical practice; clinical diagnosis guidelines should be shared between the specialists in order to improve the diagnostic and therapeutic approch of our physicians.

## Introduction

L'érysipèle est une infection cutanée aiguë d'origine bactérienne se développant dans les tissus dermiques et hypodermiques, non nécrosante et fréquemment récidivante [1]. Elle est majoritairement streptococcique [1,2]. C'est une infection communautaire fréquente qui intéresse aussi bien la médecine ambulatoire que la médecine hospitalière par le recrutement et le traitement des patients. Cependant, les publications se rapportant à sa prise en charge diagnostique et thérapeutique sont le plus souvent basées sur des études hospitalières [3,4]. De ce fait, le regard sur cette pathologie est amputé des informations qui proviennent de la pratique courante en médecine de ville, notamment de la pratique des médecins généralistes. L'objectif de notre enquête était d'évaluer la prise en charge de l'érysipèle par les médecins généralistes de Marrakech et de la confronter aux différentes recommandations et données récentes de la littérature, afin de mesurer les écarts avec celles-ci, et de proposer des solutions d'amélioration.

## Méthodes

Il s'agit d'une étude transversale à visée descriptive et analytique portant sur l'évaluation de la prise en charge de l'érysipèle par les médecins généralistes des secteurs public et privé de la ville de Marrakech au Maroc. Elle s'est déroulée du 19 Mai au 20 Octobre 2014, sous forme d'une enquête réalisée à l'aide d'un questionnaire respectant l'anonymat. Les données collectées ont été saisies et analysées au moyen du logiciel SPSS version 16 au laboratoire d'épidémiologie clinique de la faculté de médecine et de pharmacie de Marrakech. L'analyse statistique a été réalisée en deux volets: une analyse monovariée à visée descriptive avec des variables qualitatives et quantitatives et une analyse bivariée des variables qualitatives à visée analytique au moyen de tests statistiques d'hypothèse, avec un seuil de signification statistique à 5% (p<0,04). Pour l'analyse monovariée, les principales variables étudiées portant sur des données épidémiologiques générales, notamment la fréquence des cas d'érysipèle rencontrés; le diagnostic positif; et l'attitude thérapeutique. Pour l'analyse bivariée, nous avons recherché s'il existait des variables statistiquement corrélées au recours aux examens paracliniques pour poser le diagnostic positif, à l'hospitalisation systématique des cas d'érysipèle, à la prescription d'une biantibiothérapie en ambulatoire pour le traitement étiologique, et à la prescription d'anti-inflammatoires non stéroidiens (AINS) et de corticoïdes pour le traitement adjuvant.

## Résultats

Nous avons sollicité 167 médecins. Le taux de réponse global était de 87%. Il était de 88,9% pour les médecins du secteur public et de 84,7% pour ceux du secteur privé. La moyenne d'âge de nos médecins était de 49 ans avec des extrêmes de 29 et 81 ans; leur répartition selon le sexe était quasi équitable. Soixante-quatre médecins (56,1%) exerçaient dans le secteur public et 50 (43,9%) dans le secteur privé, Le nombre d'années d'exercice minimum était de 4 ans et au maximum 50 ans. Dans leur pratique, la fréquence de l'érysipèle était de 1 à 5 cas par an pour 64,9%. Les principaux facteurs favorisants généraux étaient le diabète sucré (71,9%) et l'obésité (33,3%). Les facteurs locorégionaux étaient une porte d'entrée à type de plaie cutanée non ou mal traitée (78,9%),un intertrigo inter orteil (72,8%),un ulcère de jambe (62,3%); la notion d'angines streptococciques (38,6%); l'existence d'une insuffisance veineuse chronique (36%) ou d'une insuffisance lymphatique (28,9%). Selon nos médecins, l'érysipèle se localise fréquemment au niveau du membre inférieur (93,9%). Il s'accompagne de fièvre (99,1%), de frissons (79,8%), de syndrome pseudo grippal (32,5%); avec la présence quasi constante d'un placard érythémateux-oedémateux (100%),d'une douleur locale (77,2%), d'adénite satellite (60,5%), avec bourrelet périphérique (46,5%). Soixante-seize médecins (66,7%) ont estimé que les examens paracliniques pourraient être indiqués dans la prise en charge de l'érysipèle; pour affirmer l'existence ou la décompensation de tare pour 58 (50,9%) médecins; rechercher des complications pour 42 (36,8%); ou éliminer des diagnostics différentiels pour 26 (22,8%). Vingt-deux médecins (19,3%) ont rapporté l'intérêt de ces examens pour poser le diagnostic positif; parmi eux, 19 (16,7%) demanderaient un hémogramme, 15 (13,2%) un dosage de CRP, 9 (7,9%) des prélèvements bactériologiques cutanés et 6 (5,2%) des hémocultures. Les complications générales d'érysipèle étaient le sepsis dans 86% des cas, et la dermohypodermite bactérienne nécrosante dans 64,9% des cas. Dix sept médecins (14,9%) décideraient de référer de principe tout cas d'érysipèle pour hospitalisation, quelle que soit la situation. Par contre, 97 médecins (85,1%) ont considéré que l'hospitalisation en première intention ne devrait être indiquée qu'en présence de facteurs de gravité. Parmi eux, 95 (83,3%) hospitaliseraient le malade s'il existe d'emblée des complications. Pour les 97 (85,1%) médecins qui traiteraient en ambulatoire l'érysipèle, le traitement est une monoantibiothérapie pour 82 (71,9%), par voie orale pour 59 médecins (51,7%); et à base de bétalactamines dans 95% des cas. L'amoxicilline orale a été préconisée par 21,9% des médecins. Les biantibiothérapies ont été préconisées par 15 médecins (13,2%); toutes indiquées en ambulatoire et comprenaient une bétalactamine (l'amoxicilline- ciprofloxacine et l'amoxicilline- gentamycine dans 20% des cas chacune). Soixante-deux médecins (54,4%) prescrivaient une molécule antalgique par voie orale essentiellement le paracétamol pour 59 (51,7%) médecins. Onze médecins (9,6%) prescrivaient les AINS par voie orale. La prescription de corticothérapie systémique de courte durée a été préconisée par 5 médecins (4,4%) à base de prednisolone (60 mg /j pendant 3 à 10 j). Neuf médecins (7,9%) avaient préconisé des injections d'héparine de bas poids moléculaire (HBPM) à dose préventive pendant 7à10 j dont 7 médecins de façon systématique et 2 en présence de facteurs de risque de thrombose veineuse profonde.

Les autres mesures thérapeutiques proposées étaient les soins appropriés au niveau d'une éventuelle porte d'entrée (13,2%), le repos au lit (12,3%), la surélévation du membre atteint, la contention élastique en cas d'insuffisance veineuse ou lymphatique et le lever précoce (6,1% chacun). Pour tous les 97 médecins qui prendraient en charge en ambulatoire un érysipèle typique, il était obligatoire de prévoir une consultation de suivi thérapeutique; 63 parmi eux (55,3%) la programmeraient après 48-72 heures. L'hospitalisation en deuxième intention devrait être indiquée si aggravation des signes cliniques pour 91 médecins (79,8%) ou une décompensation de tare pour 84 (73,7%) ou si la fièvre persistait malgré 72 heures d'antibiothérapie pour 50 (43,9%). Les mesures prophylactiques proposées étaient essentiellement le traitement approprié de toute effraction cutanée (94,7%), la chimioprophylaxie par benzathine-pénicilline G ou par l'érythromycine à partir de la deuxième récidive (46,5%), et le drainage manuel en cas de lymphoedème congénital ou acquis (24,6%). L'analyse bivariée avait montré que : la proportion de médecins posant le diagnostic positif d'érysipèle en se basant sur des examens paracliniques était plus élevée dans le secteur privé que dans le secteur public (p = 0,404); La proportion de médecins proposant d'hospitaliser tout cas d'érysipèle était plus élevée dans le secteur public que dans le secteur privé (p = 0,933) ainsi que chez les médecins ayant 15 ans d'activité ou moins que chez ceux ayant plus de 15 ans (p = 0,122); Une relation statistiquement significative (p = 0.001) a été mise en évidence entre la prescription d'une biantibiothérapie et l'exercice dans le secteur privé; Une relation statistiquement significative (p = 0.008) a été mise en évidence entre la prescription d'AINS en ambulatoire et l'exercice dans le secteur privé ainsi qu'à l'ancienneté d'exercice de plus de 15 ans (p = 0.028); Une relation statistiquement significative (p = 0,010) a été mise en évidence entre la prescription d'une corticothérapie systémique courte et l'exercice dans le secteur privé.

## Discussion

L'érysipèle est une maladie communautaire sans caractère épidémique et sans recrudescence saisonnière nette. C'est une pathologie dont l'incidence estimée est de 10-100 cas pour 100 000 habitants/an en France et dont la moyenne d'âge de survenue est 55-65 ans [4,5] concordant avec les résultats de notre étude. Cependant, selon Mahe et al, l'érysipèle peut atteindre des populations plus jeunes soumises à un risque particulier d'effractions cutanées récurrentes au niveau des pieds [6]. Conformément à la littérature, des facteurs de risque locaux ou généraux ont souvent été retrouvés par nos médecins en cas d'érysipèle [1,5,7-9]. Le diagnostic positif des formes typiques était clinique pour la majorité. Pour de nombreux auteurs, le regroupement des éléments sémiologiques cliniques est d'une valeur fiable pour poser le diagnostic d'érysipèle [1,2,10,11]. L'érysipèle siège dans plus de 85% des cas aux membres inférieurs, pour moins de 10% au visage et 2 à 12% aux membres supérieurs [1]. Les localisations électives désignées par nos médecins étaient le membre inférieur pour la majorité, ce qui est comparable aux résultats de l'étude de jego et al (96%) [12], de Cissé et al (90%) [13] et celle de Amal et al (87%) [14]. Les cas d'érysipèle engageant le pronostic vital sont très peu fréquents sous traitement bien conduit [1]. Les formes compliquées peuvent être générales, notamment la décompensation de tares chez des patients multitarés; qui est la plus fréquente complication et la principale cause de décès [15,16]. Le sepsis, le choc septique sont rares (2 à 5%). Ils sont à guetter chez des patients âgés, multitarés ou immunodéprimés. Dans notre étude, le sepsis (85,9%) a été la complication générale la plus fréquemment citée, alors que la décompensation de tare n'a été désignée que par 1,8%. Selon la littérature, La complication locale la plus fréquente est l'abcès (3 à 12%) des cas, d'évolution favorable après drainage, sans modification d'antibiothérapie [15,17], il a été rapporté par 26% de nos médecins et par 6,2% dans l'étude de Chakroun et al [18]; Les thromboses veineuses profondes (TVP); citées par La moitié de nos praticiens; seraient plus en rapport avec l'impotence fonctionnelle et l'alitement chez des patients préalablement à risque qu'à l'érysipèle lui-même [19]. La place des examens paracliniques devrait être bien connue pour en justifier le recours en pratique de ville. Selon nos médecins, le recours aux examens para-cliniques ne s'avèrait nécessaire que pour les formes sévères, atypiques ou compliquées. L'échographie et le scanner ainsi que l'IRM n'offrent pas de bénéfice supplémentaire pour le diagnostic d'érysipèle par manque de spécificité [1]. En cas de doute diagnostique dans les formes sévères, le recours à une IRM en urgence peut être justifié [9]. Dans notre étude, La prise en charge doit se faire en ambulatoire pour 85,1% des médecins; 14,9% des médecins opteraient pour l'hospitalisation systématique de tout érysipèle, ce qui est comparable aux 12% et 10% d'hospitalisations décidées de principe dans les enquêtes prospectives respectivement hospitalière de Jégou et al [20] et de ville de Kopp et al [21]. Par contre, dans l'enquête prospective de ville de Larivière et al [22] aucune hospitalisation n'a été décidée de principe. Comme dans notre enquête, l'hospitalisation pour cause de comorbidité a une place non négligeable dans les enquêtes de Jégou et al (53%) [69], de Kopp et al (63,1%) [21] ainsi que de Larivière et al (40%) [22] qui se sont penchées sur les motifs d'hospitalisation des érysipèles contre (80,7%) chez nous (Tableau 1). Dans les études prospectives de Larivière et al [22] et de Kopp et al [21], tous les érysipèles ont été traités par monoantibiothérapie ce qui a été préconisé aussi par 84,5% de nos médecins. La biantibiothérapie a été prescrite par 15,5% malgré que ces associations pourraient théoriquement contribuer à l'émergence des souches bactériennes résistantes.

**Tableau 1:** Motifs d’hospitalisation initiale de l’érysipèle dans différentes études

Motifs d’hospitalisation	Jégou et al[20]E. prospective hospitalière	Larivière et al[22]E. prospective de ville	Kopp et al[21]E. prospective de ville	Kopp et al[21]E. déclarative de ville	Notre enquête
Systématique	12	0	10,5	-	14,9
Sévérité du tableau clinique	-	-	-	64,2	55,3
Comorbidité	53	40	63,1	51,1	80,7
Difficulté d’observance à domicile/contexte social	7	-	15,8	4,4	71,9
Existence ou suspicion de complication	-	40	-	13,9	83,3

L'amoxicilline a été la plus fréquemment proposée (21,9%) par nos médecins de même qu'elle a été la plus fréquemment prescrite dans l'étude de Kopp et al (29,3%) [21]. Chez Larivière et al (18,4%) [22], elle se place en troisième position par ordre de fréquence de prescription. Les modalités de prescription de nos enquêtés étaient en phase avec les recommandations de la Société Française de Dermatologie (SFD) et de la Société de Pathologie Infectieuse de Langue Française (SPILF 2000). L'amoxicilline-acide clavulanique occupait la deuxième position aussi bien dans notre enquête (20,2%) que dans celle de Larivière et al (26,5%) [22]. Par contre, la pristinamycine qui représentait la plus grande proportion des prescriptions chez Larivière et al (30,6%) [22] et qui vient en seconde position chez Kopp et al (26,7%) [21] ne constituait pas vraisemblablement un recours habituel dans la pratique de nos enquêtés (2,6%) vu sa disponibilité irrégulière (Tableau 2). Partant des résultats de notre enquête ainsi que celle de Larivière et al [22], il s'avère important de sensibiliser les praticiens au recours à l'amoxicilline pour les érysipèles pris en charge en ville, afin de réserver l'usage de l'amoxicilline protégée pour des affections mieux circonscrites par son spectre plus élargi. Bien que très souvent cité dans l'arsenal thérapeutique de l'érysipèle par plusieurs traités de médecine et recommandé par la conférence de consensus en cas de fièvre mal tolérée [1], aucune étude clinique ne s'est attelée à décrire la fréquence et les modalités du recours aux antalgiques en cas d'érysipèle dans les limites de notre recherche. Un traitement antalgique a été préconisé par 63,9% des enquêtés. Dans notre enquête, 9 médecins (7,9%) ont préconisé des injections d'HBPM à dose préventive pendant que Larivière et al [22] et Kopp et al [21] ont retrouvé respectivement 13% et 45,9% de recours systématique à l'anticoagulation préventive. la SFD et la SPILF [1] recommandent de considérer le risque thromboembolique pour chaque patient avant de recourir à la réalisation d'un écho-doppler veineux ou à la prescription d'une anticoagulation préventive.

**Tableau 2:** Antibiotiques préconisés dans différentes études pour traiter l’érysipèle

Molécules antibiotiques (%)	Voie d’administration	Larivière et al [22]	Kopp et al [21]	Notre enquête
Pénicilline G	Injectable	-	-	14,9
Pénicilline G retard	Injectable	-	-	2,6
Pénicilline V	Orale	-	13,3	3,5
Pénicilline G relais Pénicilline V	Injectable/orale	-	-	1,7
Pénicilline G relais Pénicilline A	Injectable/orale	-	-	0,9
Pénicilline M (Flucloxacilline)	Orale	18,4	16	2,6
Pénicilline A (Amoxicilline)	Orale	18,4	29,3	21,9
Pénicilline A protégée (Amox - Ac. clav)	Orale	26,5	-	20,2
Pristinamycine	Orale	30,6	26,7	2,6
Autres macrolides ou apparentés	Orale	2	4	-
Ciprofloxacine	Orale	-	6,7	0,9
Ac. Fucidique	Orale	2	2,7	-

Les taux de prescriptions des AINS/corticoïdes dans l'enquête prospective de Larivière et al [22] (12%) est comparable à celui des déclarations favorables à ces thérapeutiques dans notre enquête. En effet, l'usage des anti-inflammatoires dans le traitement de l'érysipèle a été un des point-clés des divergences dans la prise en charge, entre leur bénéfice thérapeutique et leur risque iatrogénique. Plusieurs publications dont la compilation Chosidow et al [23], citée par la SFD et la SPILF [1], avaient fait état de survenue de fasciites nécrosantes lors de l'usage des AINS au cours de DHBNN. La balance entre risque potentiel important et bénéfice potentiel minime a conduit la conférence de consensus de la SFD et la SPILF à déconseiller l'usage des agents anti-inflammatoires dans le traitement des DHBNN. Selon la SFD et la SPILF, la surélévation du membre atteint, la mise en place d'une contention veineuse et le lever précoce sont à favoriser chez les malades à risque de développer une TVP, bien que n'ayant jamais été évalués dans le cadre du traitement de l'érysipèle [1]. Ils ont été proposés par 6,1% de nos enquêtés chacun, alors qu'ils n'ont pas été mentionnés ni par Larivière et al [22] ni par Kopp et al [21]. Pour s'enquérir de la bonne évolution sous traitement, une consultation de contrôle s'impose dans les 48-72 heures de début du traitement à domicile selon la conférence de consensus. Tous nos enquêtés favorables à la prise en charge de l'érysipèle typique en ambulatoire l'ont également prévue. L'hospitalisation devrait être décidée au cours de cette visite en cas de persistance de la fièvre, d'aggravation clinique locale ou générale et de décompensation d'une maladie sous-jacente [1]. Ces assertions rencontraient respectivement 43,9; 79,8 et 73,7% des avis favorables de nos enquêtés. Les préventions primaires et secondaires ont rencontré l'intérêt de nos médecins dont 94,7% ont été favorables au traitement des portes d'entrée cutanées et 46,5% à l'antibioprophylaxie à partir de la deuxième récidive ce qui concorde avec les données de la littérature [1, 24].

## Conclusion

IL ressort de cette enquête que les connaissances de nos médecins sont en phase avec les données récentes de la littérature concernant le diagnostic d'érysipèle, mais que des efforts doivent être fournis pour vulgariser celles qui concernent le traitement afin d'optimiser leurs pratiques professionnelles. Pour se faire; nous suggérons: la réalisation d'études descriptives qui permettraient de mieux situer l'érysipèle en termes d'épidémiologie descriptive et causale ainsi que d'études prospectives analysant les pratiques réelles des médecins dans notre contexte. Ceci requerra certes des moyens logistiques plus importants et pourquoi pas l'implication d'organismes voués à la gestion des soins ambulatoires dans la région de Marrakech. La réalisation d'études prospectives comparant différentes thérapeutiques, notamment antibiotiques, selon l'épidémiologie locale afin de ressortir des recommandations de pratiques professionnelles marocaines actualisées.

### Etat des connaissances actuelle sur le sujet

- L'érysipèle est une infection dermo-épidermique fréquente de prise en charge en ambulatoire ou en milieu hospitalier;
- L'érysipèle nécessite une bonne prise en charge diagnostique et thérapeutique afin d'éviter des complications éventuelles;
- Les données de la littérature se rapportant à sa prise en charge diagnostique et thérapeutique sont le plus souvent basées sur des études hospitalières.

### Contribution de notre étude à la connaissance

- Contribuer à la mise en lumière des aspects méconnus de l'érysipèle en médecine de ville à travers une description à l'échelle de la ville de Marrakech, des pratiques professionnelles des médecins généralistes en ce qui concerne le diagnostic, le traitement et la prévention de l'érysipèle;
- L'étude approuve la concordance des pratiques des médecins généralistes avec les recommandations en matière de diagnostic, cependant, une amélioration des pratiques thérapeutiques sont nécessaires;
- Des études approfondies sont nécessaires sur ces pratiques professionnelles pour une meilleure prise en charge de cette infection.

## Conflits d’intérêts

Les auteurs ne déclarent aucun conflit d'intérêts.
